# Enhancing fundus image analysis for diabetic retinopathy using CheXNet with CBAM and Grad-CAM visualization

**DOI:** 10.3389/fmed.2026.1732109

**Published:** 2026-02-25

**Authors:** Wedad Al-Dolat, Salem Alhatamleh, Noor Alqudah, Amro Alhazimi, Mohammad Amin, Aseel Daamseh, Rola Madain, Raghad Malkawi, Rami Al- Omari, Faisal Almarek, Sarah Husam Aljefri

**Affiliations:** 1 Department of Ophthalmology, Faculty of Medicine, Yarmouk University, Irbid, Jordan; 2 Computer Science Department, Faculty of Information Technology and Computer Sciences, Yarmouk University, Irbid, Jordan; 3 Department of Ophthalmology, Faculty of Medicine, Jordan University of Science and Technology, Irbid, Jordan; 4 Department of Ophthalmology, College of Medicine, Imam Mohammad Ibn Saud Islamic University (IMSIU), Riyadh, Saudi Arabia; 5 Faculty of Medicine, Jordan University of Science and Technology, Irbid, Jordan; 6 Department of Obstetrics and Gynecology, Faculty of Medicine, Jordan University of Science and Technology, Irbid, Jordan; 7 Department of Pharmacy, Jordan University of Science and Technology, Irbid, Jordan

**Keywords:** deep learning, diabetic retinopathy, fundus imaging, Grad-CAM, image classification

## Abstract

**Introduction:**

Diabetic retinopathy (DR) is a leading cause of vision impairment among individuals with diabetes. Early detection and accurate grading are essential for timely clinical management. However, developing robust models for automated interpretation and grading of fundus images remains challenging due to variability in lesion appearance and image quality.

**Methods:**

This study proposes a deep learning framework for DR classification from fundus images based on a DenseNet121 backbone initialized with CheXNet weights. A Convolutional Block Attention Module (CBAM) is integrated to enhance feature representation through channel and spatial attention mechanisms in a data-driven manner. In addition, Gradient–weighted Class Activation Mapping (Grad–CAM) is employed to provide post hoc visual explanations of model predictions. The proposed CheXNet_CBAM model is evaluated against several convolutional neural network architectures, including CheXNet, DenseNet121, MobileNetV2, VGG19, and ResNet50, using the APTOS 2019 and DDR datasets.

**Results:**

On the APTOS 2019 dataset, the proposed model achieves an accuracy of 96.12%, while on the DDR dataset it attains 96.33%, outperforming the compared architectures on both benchmarks.

**Discussion:**

The results indicate that incorporating CBAM improves discriminative feature learning within a DenseNet121–based framework. While the model demonstrates strong performance across two public datasets, further prospective evaluation and external validation are required to assess its clinical applicability in real–world settings.

## 1 Introduction

Diabetes mellitus (DM) describes a group of chronic endocrinological disorders characterized by sustained hyperglycemia in untreated individuals (1). A total of 415 million people between the ages of 20 and 79 were estimated to be suffering from the disease in 2015, 5 million of whom are estimated to have lost their lives from diabetic complications in that same year, and historical data show a steady increase in incidence rate as the years pass by and the global population increases (2).

Diabetic retinopathy (DR) is a neurovascular complication of diabetes that damages the microvasculature in the retina. Endothelial cells, gliocytes, pericytes, and white blood cells are among the various cell types affected by sustained high blood glucose levels. Therefore, leading to changes in the permeability of small blood vessels and blood perfusion levels to the retina, causing retinal leakage and retinal ischemia (3).

DR remains the primary cause of vision loss among the working-age population. Worldwide, DR ranked as the fifth most common cause of both preventable blindness and moderate to severe visual impairment between 1990 and 2010 (4). It's diagnosed based on characteristic vascular abnormalities seen during clinical examination of the eye. In 1968, the Airlie House classification was first introduced and is considered the basis of all modern DR classification systems. This classification categorizes DR into non-proliferative diabetic retinopathy (NPDR) and proliferative diabetic retinopathy (PDR) (5). NPDR features two main vascular changes in the retina: elevated permeability and capillary occlusion. During this phase, microaneurysms, hemorrhages, and hard exudates are observed by fundus photography (6). Proliferative diabetic retinopathy, the more advanced phase, is characterized by abnormal growth of new retinal vessels. Most patients during this stage experience vision impairment due to retinal detachment or vitreous hemorrhage (7).

Slight modifications were made to the Airlie House classification in 1991 for the Early Treatment of Diabetic Retinopathy Study (ETDRS). The ETDRS severity scale is widely used in clinical research and trials to predict progression and evaluate treatment plans (8). The retinopathy severity level was assessed at the eye level, with 14 levels ranging from level 10 (No DR) to level 85 (PDR) (5). Starting with (level 10–13), known as no diabetic retinopathy, the retina is healthy with no evidence of microaneurysms or hemorrhages. Followed by mild non-proliferative retinopathy (level 14–20) that is characterized by microaneurysms only. On the other hand, moderate non-proliferative retinopathy (levels 35–53) is characterized by the presence of two or more of the following features, such as microaneurysms, hemorrhages, and hard exudates. Severe non-proliferative retinopathy (level 53–61) is marked by 20 hemorrhages in each of the four quadrants, venous beading in two quadrants, or intraretinal microvascular abnormalities in one quadrant. The most advanced stage is proliferative diabetic retinopathy (level 61–85), where the vasoproliferative factors produced by the retina trigger neovascularization, often leading to vitreous hemorrhage (9).

The type of DM influences the occurrence and progression of DR. 10%−15% of diabetics are of type 1, whereas the remaining patients are type 2 diabetics. Within 10 years, 71%−90% of patients with type 1 diabetes and 67% of type 2 diabetes will develop DR. Thus, screening is highly significant throughout the disease's long latent phase to prevent vision loss (10).

Color fundus photography (CFP) has been acknowledged as the gold standard for the screening and analysis of diabetic retinopathy (DR), while dilated fundus examination is the mainstay of early diagnosis. Several major organizations, including the American Academy of Ophthalmology, the American Diabetes Association, and the Canadian Ophthalmological Society, released screening guidelines stating that patients with type 2 DM should have a dilated fundus examination at the time of diagnosis, while those with type 1 DM should have their first examination 5 years after diagnosis. In both cases, yearly follow-up examinations are suggested (11). Although there are still measures taken to inform patients and doctors about the situation, a significant portion of the patients who are supposed to get treated medically remain untreated because of the reasons such as poor compliance or lack of access to screening for retinal diseases (12). With traditional techniques to determine DR often being slow, needing specific clinical inpatient appointments, and making the patient wait for a prolonged period to get prepared are the reasons why such diagnoses are sometimes delayed or the patients have no access to screenings (13).

As diabetes incidence approaches pandemic levels, implementing advanced cost-effective methods for early detection of DR is essential (13). One promising approach is the use of AI models. AI has evolved over the years from basic experimental models to systems used daily in healthcare settings (14). Processing and evaluating large quantities of retinal images is difficult due to early detection concerns; these activities usually require higher accuracy and robustness. According to studies, these disadvantages have shifted the focus of research toward deep learning-based automated detection systems. Deep learning is increasingly being used to solve problems with medical image classification. Deep convolutional neural networks (DNNs) outperform other computer vision techniques. Over the last decade, numerous scholars have developed unique DNN architectures for picture categorization. MobileNet (15), VGGNet, ResNet50, InceptionNet, and XceptionNet (16), many other models are popular.

The goal of this study is to use deep learning techniques based on the APTOS 2019 and DDR datasets to create a fully automated system for diagnosing the severity of diabetic retinopathy. The primary contributions of the suggested model are as follows:

- This study introduces a new hybrid model, CheXNet_CBAM, for detecting and distinguishing diabetic retinopathy from normal fundus images.
- Use of Gradient Maps (Grad-CAM) to illustrate the regions most influential in the classification process, enhancing the interpretability of the model.
- Achieving high performance on diverse datasets (96.12% on APTOS-2019 and 96.33% on DDR) demonstrates the effectiveness of the model in supporting early diagnosis and clinical practice.
- This approach achieves superior diagnosis accuracy compared to other state-of-the-art methods, including CheXNet, DenseNet121, MobileNetV2, VGG19, and ResNet50 models.
- The study showed that the proposed model outperforms most previous models and methods in the field of diabetic retinopathy diagnosis, in terms of accuracy and ability to focus on clinically important areas.

In Section 2, the methods used are discussed, followed by an extensive discussion on the dataset, and the last part is a sketch of the proposed approach and training techniques. Section 3 performs data analysis and rates the proposed model as to its effectiveness in the diabetic retinopathy diagnostic tests. Section 4 highlights the most significant research in the field of diabetic retinopathy diagnosis and contrasts it with the proposed model. Finally, Section 5 wraps up the paper with conclusions and suggestions for future research.

## 2 Methodology

**Figure 1:**
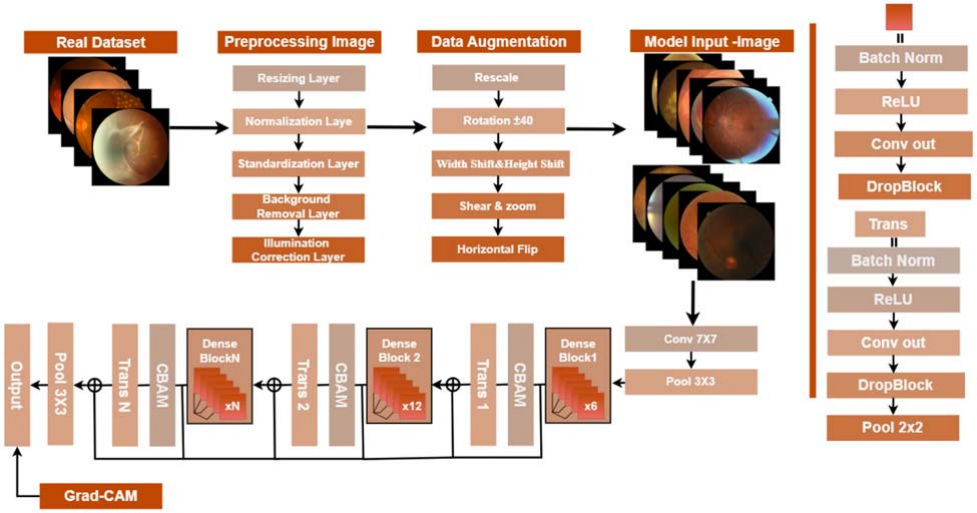
The complete architecture of the CheXNet_CBAM model for diagnosing diabetic retinopathy.

A deep learning framework is put forth in this research, which classifies diabetic retinopathy (DR) automatically into five levels of severity, namely: No DR, mild, moderate, severe, and proliferative. The model proposed, CheXNet_CBAM, depicted in Figure 1 is a modification of the architecture of DenseNet121. It imitates the configuration of CheXNet for examining chest X-ray images and modifies it for retinal fundus imaging. To improve the representation of features, we add a convolutional block attention module (CBAM), which integrates channel and spatial attention, thus allowing the network to concentrate more on the retinal regions that are clinically important. To make the model more robust and at the same time to further reduce the likelihood of overfitting, the DropBlock algorithm is used, which is a technique to drop contiguous patches of feature maps instead of just isolated pixels. The Transformer encoder layers are placed after the convolutional backbone to effectively model long-range dependencies, capturing thus both local textures and global structural patterns. Class-weighted loss functions prevent existing imbalances in the dataset, working effectively with the aggressive data augmentation techniques such as rotation, brightness changes, and zooming employed. Among the key hyperparameters are the learning rate, dropout value, and block size, all thoroughly tuned to guarantee stable, effective training. For interpretation, we use an improved Grad-CAM visualization to highlight retinal regions used in the model prediction. These improvements-CBAM, DropBlock, hybrid CNN-Transformer architecture, and explainable visualizations to a huge leap in accuracy, generalization, and clinical dependability over and above the baseline CheXNet model.

### 2.1 Dataset acquisition

The dataset considered in this study consists of retinal fundus images, which were pre-processed with an isotropic Gaussian filter to remove noise and improve clarity. The original dataset was obtained during the APTOS 2019 Blindness Detection challenge (17). Each image was labeled with the severity of diabetic retinopathy (DR) via the train.csv file that came along with the original dataset. Images are arranged into five separate folders, each corresponding to one DR stage. The class-wise distribution is given in the Table 1.

**Table 1:** Distribution of retinal images across diabetic retinopathy severity levels for the APTOS 2019 dataset.

**Class (Severity)**	**Number of images**
No_DR (No Diabetic Retinopathy)	1,805
Mild	370
Moderate	999
Severe	193
Proliferate_DR (Proliferative DR)	295
**Total**	**3,662**

Bold value (Total = 3,662) indicates the aggregate sum of the dataset distribution.

**Figure 2:**
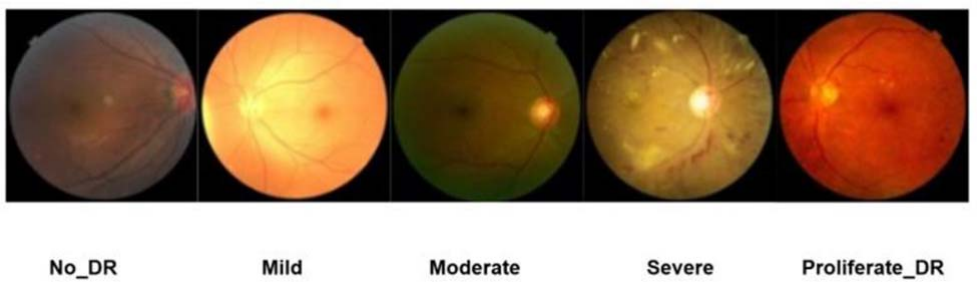
Example of each class of the data set.

The Diabetic Retinopathy (DDR) dataset comprises 13,673 retinal fundus images collected from 147 hospitals across 23 provinces in China (18). Each image is assigned one of five classes depicting diabetic retinopathy (DR) severity: No_DR, Mild, Moderate, Severe, and Proliferative_DR. The original dataset included a sixth category corresponding to poor-quality images, which were excluded in this study to ensure reliable training. This brings the total number to 12,522 images. Also, all of the images were preprocessed to remove the black background since it boosted the visibility of retinal structures and would have taken away from irrelevant information. The distribution of images in the five DR severity levels is given in Table 2. Figure 2 shows fundus images from the dataset.

**Table 2:** Distribution of images in the DDR dataset.

**Class (Severity)**	**Number of images**
No_DR (No Diabetic Retinopathy)	6,266
Mild	630
Moderate	4,477
Severe	236
Proliferate_DR (Proliferative DR)	913
**Total**	**12,522**

Bold value (Total = 12,522) indicates the aggregate sum of the dataset distribution.

Potential confounding factors such as patient age, gender, geographic region, and imaging device variability were addressed at the dataset acquisition level. The DDR dataset was collected from 147 hospitals across 23 provinces using standardized acquisition principles and diverse fundus camera types, with balanced demographic representation. This multicenter and multi-device design reduces systematic bias and improves generalizability. In both the DDR and APTOS 2019 datasets, diabetic retinopathy severity labels serve as the primary stratification variable, reflecting clinically meaningful disease progression. As the study is image-centered and does not involve patient-level intervention or outcome modeling, statistical confounding adjustment methods such as propensity score matching are not directly applicable.

The sample size used in this study was determined by the availability of publicly accessible benchmark datasets, namely APTOS-2019 and DDR, which are widely adopted in diabetic retinopathy research. As this work follows a data-driven deep learning framework rather than a hypothesis-testing statistical design, conventional sample size calculation, effect size estimation, and power analysis are not directly applicable. Model validity is instead assessed through generalization performance on independent test sets and consistent comparative evaluation across multiple architectures and datasets. This approach is standard practice in contemporary medical image analysis research.

### 2.2 CheXNet model structure

CheXNet refers to a deep CNN model designed specifically for the automated identification of pneumonia and thoracic illnesses from chest X-ray images (19). The model is based on DenseNet121, a 121-layer deep architecture, which introduces dense connections between all the layers and their preceding layers. This direct linkage of layers enables a layer to access the feature maps of all previous layers at once, which improves the gradient flow, promotes reuse of features and has fewer parameters than a conventional deep CNN of the same depth. This architecture helps a lot with medical imaging since it recognizes very subtle patterns at multiple scales. DenseNet121 is a construction of dense blocks with transition layers, such that in a dense block, each layer takes the concatenation of the feature maps of all preceding layers as input. Formally, for a layer *l* with input feature maps *x*_0_, *x*_1_, …, *x*_*l*−1_, the output *x*_*l*_ is:


$$\begin{array}{l} x_{l}=H_{l}\left(\left[x_{0},x_{1},\ldots ,x_{l-1}\right]\right) \end{array}$$ (1)

Where ([*x*_0_, *x*_1_, …, *x*_*l*−1_]) is the concatenation operator and *H*_*l*_(·) is a composite function of Batch Normalization, ReLU activation, and a 3 × 3 convolution. Thus, this setup allows the network to blend low- and high-level features efficiently to pick up subtle patterns in medical images. Transition layers lie between dense blocks to shrink the size of feature maps and reduce channel numbers to increase computational efficiency and counter overfitting. Each transition layer carries out 1 × 1 convolution with 2 × 2 average pooling as follows:


$$\begin{array}{l} x_{\text{transition}}=AvgPool_{2\times 2}\left(Conv_{1\times 1}\left(x_{dense}\right)\right) \end{array}$$ (2)

**Figure 3:**
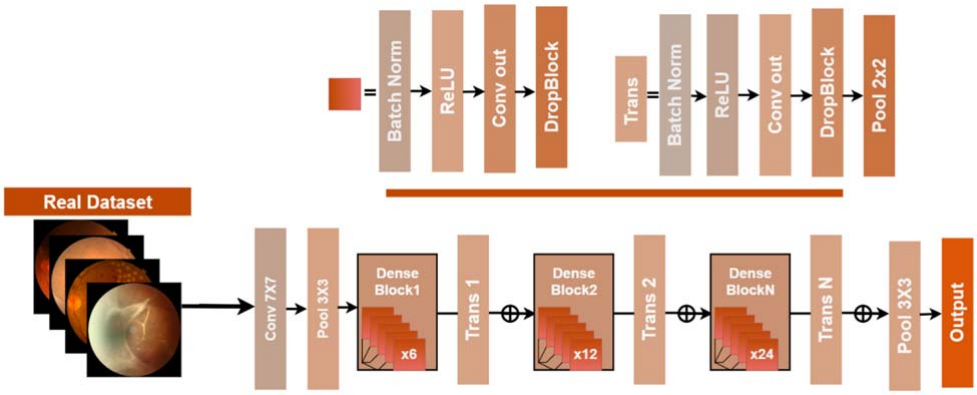
Hierarchical structure of the CheXNet model with details of condensed blocks and processing layers.

After the final dense block, CheXNet applies Global Average Pooling (GAP) to reduce each feature map to a single value (20). The resulting vector is then passed through a fully connected layer with sigmoid activation for multi-label classification:


$$\begin{array}{l} {ŷ}^{i}=\sigma \left(w_{i}^{T}x_{GAP}+b_{i}\right)\;,\;i=1,2,\cdots \;,C \end{array}$$ (3)

where *C* is the number of disease classes, $w_{i}^{T}$and *b*_*i*_ are learnable weights and biases, respectively, and σ(·) gives the probability of each class between 0 and 1. CheXNet is trained by means of binary cross-entropy loss so that the network can output probabilities closely reflecting actual presence and absence of each disease. There are a number of limitations of the CheXNet though. It belongs to the class of domain-specific algorithms because it has been originally trained on chest X-ray images; thus, direct application to other modalities, such as retinal images, is suboptimal. DenseNet (21), on the other hand, mainly extracts local features and thus it cannot maximally utilize the long-range spatial relation between features. Either way, CheXNet, with its dense connectivity, multi-scale feature reuse, and deep design, provides a very good backbone for analysis of medical images. Nonetheless, applying it to a new domain usually requires modifications to the architecture in terms of attention modules, stronger regularization, and improved interpretability techniques. In short, to make CheXNet work for retinal fundus images, it must be channeled to outweigh certain clinically relevant eye regions and to capture long-range dependencies. Hence, attention modules, hybrid CNN-transformer architectures, and other specialized training techniques are some of the improvements that must be considered for reliable classification of eye diseases. These improvements and architectural changes are described in detail in the next section. Figure 3 shows the hierarchical structure of the CheXNet model.

Although CheXNet was originally proposed for chest X-ray image analysis, in this work, it is adapted to the retinal fundus image domain through transfer learning and architectural modification. The DenseNet121 backbone of CheXNet is initialized using pretrained weights, while the original classification layer—designed for thoracic disease detection—is removed and replaced with a task-specific fully connected layer tailored to diabetic retinopathy classification. The network is then fine-tuned on retinal fundus datasets, allowing the learned low-level and mid-level visual representations to be adjusted to the distinct texture, color distribution, and lesion patterns of fundus images. Furthermore, to mitigate the domain gap between chest X-ray and retinal imaging modalities, a Convolutional Block Attention Module (CBAM) is incorporated into the architecture. This enables the model to emphasize clinically relevant retinal structures such as microaneurysms, hemorrhages, and exudates, while suppressing irrelevant background information. Through this adaptation strategy, CheXNet serves as a robust and transferable backbone rather than a domain-restricted model, making it suitable for accurate diabetic retinopathy diagnosis.

Unlike existing diabetic retinopathy studies that primarily apply attention mechanisms directly to generic DenseNet-based architectures or design task-specific CNNs from scratch, this work introduces a novel adaptation of the CheXNet architecture—originally developed and validated for chest X-ray analysis—into the retinal fundus imaging domain. The novelty of the proposed CheXNet_CBAM model lies in three key aspects. First, it repurposes CheXNet as a medical-image backbone beyond thoracic imaging and systematically enhances it with a convolutional block attention module (CBAM) to address the unique spatial and pathological characteristics of retinal fundus images. Second, the integration of CBAM enables joint channel-wise and spatial attention within a densely connected architecture, allowing the model to emphasize clinically relevant retinal regions (e.g., microaneurysms, hemorrhages, and exudates) without introducing additional supervision or lesion annotations. Third, the proposed framework is extensively validated across two heterogeneous public datasets (APTOS-2019 and DDR), demonstrating strong cross-dataset generalization, which is rarely examined in prior attention-augmented DenseNet or CheXNet-based DR studies. Furthermore, the combined use of CBAM with Grad-CAM visualization enhances model interpretability, providing clinically meaningful explanations that support practical deployment. Together, these contributions distinguish the proposed CheXNet_CBAM model from existing attention-based DR approaches and highlight its incremental methodological and clinical value.

### 2.3 Enhancements on CheXNet

In the proposed model, we highlight a number of modifications that have to do with CheXNet in such a way that it becomes a suitable tool for retinal fundus image analysis. As a first step, various data preprocessing and augmentation techniques have been performed to alleviate the problems caused by the variability of images and the imbalance of classes. After that, a Convolutional Block Attention Module (CBAM) is added to the model to inform the network of clinically significant retinal areas. To conclude, DropBlock regularization is introduced as a means of preventing overfitting and improving the model's capability of generalization. Additionally, Transformer encoder layers are added after the feature extraction from convolutional networks to allow the model to capture long-range dependencies and global structural patterns. The learning rate, dropout, and block size are among the hyperparameters whose careful tuning ensures the training stability. Lastly, for the sake of model interpretability, enhanced Grad-CAM visualization is employed to delineate regions that are specific to the lesion, and this, in turn, provides clinical transparency. In total, these changes raise the baseline CheXNet applied to retinal disease classification in terms of accuracy, robustness, and reliability to a considerable extent.

#### 2.3.1 Data preprocessing and augmentation for imbalanced datasets

Images in the retinal fundus dataset were resized to 224 × 224 pixels in order to get them ready for model training. The input resolution needed by DenseNet121 (22) was fulfilled this way. Normalization of pixel intensities was performed so that all the pixel values fell within the range of 0–1; then, they were standardized to have zero mean and unit variance. This was expressed as:


$$\begin{array}{l} {\text{x}}^{\prime }=\frac{\text{x}-\text{μ}}{\text{σ}} \end{array}$$ (4)

x stands for the original pixel value, μ stands for the mean of the dataset, and σ stands for the standard deviation. This preprocessing step not only reduces light variability but also improves contrast and hence, more stable and effective feature extraction is possible (23). To deal with the issue of class imbalance in the dataset, extensive augmentation techniques were used. Random geometric transformations, including rotation, scaling, and horizontal flipping, mimicked changes in patient positioning, meanwhile photometric adjustments, like brightness and contrast jittering, took care of the variations in imaging conditions. Brightness adjustment can be defined mathematically as:


$$\begin{array}{l} {\text{I}}^{\text{′}}=\text{αI}+\text{β} \end{array}$$ (5)

*I* stands for the original image, α is the parameter for controlling contrast and β is for adjusting brightness. Implementation details (as used in this study). In practice, augmentation was implemented using Keras Imag eData Generator to improve robustness to acquisition and positioning variability. The applied transformations were random rotations (up to 40°), width and height shifts (up to 0.2), shear (up to 0.2), zoom (up to 0.2), horizontal flipping, and nearest-neighbor filling. These geometric transformations enhance generalization by simulating plausible variations in imaging conditions and patient positioning. In addition, photometric variations (e.g., brightness/contrast changes) are conceptually captured by Equation 5 and can be applied through brightness/contrast jittering within the augmentation policy when needed.

Besides the augmentation, the classes' sample sizes were equalized by means of specific oversampling and dataset balancing, which made it possible for all classes to have almost the same number of images (24). Concretely, we constructed a balanced dataset using random oversampling with replacement, where minority-class images were duplicated until each class matched the number of samples in the majority class. This method of balancing has the effect of giving the model the same opportunity to experience all the severity levels thus reducing the bias toward the majority classes and enhancing the classification performance on the underrepresented categories.

##### 2.3.1.1 Balancing statistics (APTOS 2019)

The original APTOS dataset was imbalanced; therefore, we balanced it by oversampling all classes to match the majority class size. After balancing, each class contained 1,805 images, yielding a total of 9,025 images (five classes). The balanced dataset was then split into 80% training, 10% validation, and 10% testing, resulting in Train = 7,220, Validation = 902, and Test = 903 samples.

##### 2.3.1.2 Balancing statistics (DDR)

The original DDR class counts were: No_DR = 6,266, Mild = 630, Moderate = 4,477, Severe = 236, and Proliferate_DR = 913 (total = 12,522). After oversampling, each class contained 6,266 images, yielding a balanced dataset of 31,330 images. The balanced dataset was then split into 80% training, 10% validation, and 10% testing, resulting in Train = 25,064, Validation = 3,133, and Test = 3,133 samples. Evaluation consistency (confusion matrix). All reported confusion matrices were computed exclusively from the held-out test split of the balanced dataset (i.e., the 10% test partition after balancing), and their row/column totals match the corresponding test split class counts. This ensures internal consistency between the dataset split used for evaluation and the reported confusion matrix values. Furthermore, class-weighted loss was incorporated during training to penalize misclassification of minority classes more heavily.


$$\begin{array}{l} x^{\prime }=\frac{N}{C\cdot n_{i}} \end{array}$$ (6)

*N* stands for the entire training sample count, *C* denotes the class quantity, and *n*_*i*_ refers to the sample count of class *i*. This method not only balances the learning process throughout all the severity levels but also reduces the negative effect of the dataset imbalance on the model's performance (23). All these steps together with the preprocessing and augmentation strategies like image resizing, normalization, photometric and geometric transformations, class balancing, and class-weighted loss as well, talk in favor of the model's learning ability to get hold of strong feature representations, generalization to untouched data, and precise classification of diabetic retinopathy severity levels.

The datasets used in this study contain complete retinal fundus images with corresponding labels, and no missing pixel-level or annotation data were encountered. Consequently, no image exclusion or imputation procedures were applied. Variability in image quality and acquisition conditions was addressed implicitly through normalization, standardization, and extensive photometric and geometric augmentation, enabling the model to learn robust feature representations without introducing imputation-related bias.

#### 2.3.2 CBAM attention module

CBAM was integrated after convolutional feature extraction to improve the model's ability to focus on clinically relevant regions of retinal images (25). Attention is applied sequentially in CBAM; thus, channel attention and then spatial attention are applied. The informative regions are emphasized while the uninformative ones are suppressed, thereby making the features more representative.

##### 2.3.2.1 Channel attention

Based on channel dimension, the channel attention modulates each feature map's importance. Given an input feature map *F*∈ℝ^*H*×*W*×*C*^, the channel attention *M*_*c*_(*F*) is computed, where *AvgPool*(*F*) and *MaxPool*(*F*) denote global average pooling and global max pooling across spatial dimensions, respectively (26), with *MLP*(·) representing a shared multi-layer perception with a hidden layer reduction ratio of *r*, and σ(·) is the sigmoid activation function. The refined feature map is acquired by multiplying the channel attention back to the original feature map:


$$\begin{array}{l} M_{c}\left(F\right)=\;\sigma \left(MLP\left(AvgPool\left(F\right)\right)+\;MLP\left(MaxPool\left(F\right)\right)\right) \end{array}$$ (7)

$$\begin{array}{l} F^{\prime }=\;M_{c}\left(F\right)⊙\;F \end{array}$$ (8)

where ⊙ denotes element-wise multiplication (27). This operation allows the network to focus on channels that carry discriminative information for diabetic retinopathy classification.

##### 2.3.2.2 Spatial attention

In the channel attention phase, the spatial attention module enfolds crucial regions in the feature maps (28). The spatial attention works by pooling the channel information via average and max pooling. The results are concatenated and passed through a convolution followed by activation via a sigmoid function, where *f*^7 × 7^ represents the convolution operation with a kernel of size 7 × 7, and ·; · denotes concatenation along the channel dimension. Thus, the final output of CBAM is:


$$\begin{array}{l} M_{s}\left(F^{\prime }\right)=\;\sigma \left(f^{7\times 7}\left(\left[AvgPool\left(F^{\prime }\right);\;MaxPool\left(F^{\prime }\right)\right]\right)\right) \end{array}$$ (9)

$$\begin{array}{l} F^{\prime \prime }\;=\;M_{s}\left(F^{\prime }\right)\;⊙\;F^{\prime } \end{array}$$ (10)

**Figure 4:**
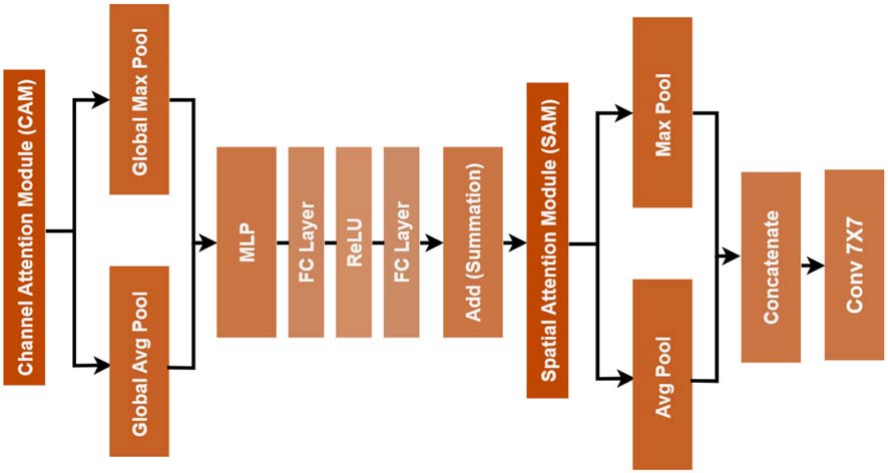
CBAM Module Architecture: Combining Channel Attention and Spatial Attention for Optimization.

With channel-spatial attention in a sequential manner, the network is directed toward both informative feature channels about spatial regions (like lesions or microaneurysms) to enhance the discriminative power of features for the different levels of DR severity. In the form of combining DenseNet121 with CBAM (29), then, the model can learn where it will look in the retinal fundus images for improved sensitivity to subtle pathological patterns while preventing irrelevant information from the background. This structure, along with data preprocessing as well as augmentation strategies, strengthens the representational power and clinical reliability of the model. The Figure 4 represents the geometry of the CBAM unit.

#### 2.3.3 DropBlock regularization

To enhance model robustness and avoid overfitting, DropBlock regularization was applied to the convolutional feature maps (30). In contrast to dropout, which zeroes out randomly chosen neurons, DropBlock sets to zero contiguous regions in the feature maps, thus forcing the network to learn robust features using distributed representations. For a feature map *F*∈ℝ^*H*×*W*×*C*^, DropBlock generates a binary mask *M*∈{0, 1}^*H*×*W*×*C*^ in which contiguous blocks of size *s*×*s* are set to zero with probability γ. The modified feature map.


$$\begin{array}{l} F^{\prime }=\frac{\left(F\;⊙\;M\right)}{mean\left(M\right)} \end{array}$$ (11)

Where ⊙ signifies multiplication over one single entry, which normalizes through mean (M) the expected activation amount across layers (31), while the probability γ of the block is calculated to represent dropout times as expected according to the size of the feature map.


$$\begin{array}{l} \gamma \;=\;\frac{\rho \;\cdot \;H\;\cdot \;W}{\left(s^{2}\cdot \;\left(H\;-\;s\;+\;1\right)\left(W\;-\;s\;+\;1\right)\right)\;} \end{array}$$ (12)

H and W are the height and width of the feature map, respectively, and s is the block size. This formulation ensures that the dropout probability scales with the spatial dimensions and block size. By dropping out contiguous regions, DropBlock encourages the network to extract redundant and distributed feature representations instead of focusing specifically on certain localized features. This is especially helpful in retinal fundus images since lesions and pathological patterns can vary in their location, size, and intensity. Therefore, beyond DropBlock's work of enhancing a model's ability to generalize from one image to another, it works to curb over-sensitivity to noise or irrelevant patterns (32). Together with CBAM and the data augmentation strategy, the DropBlock method builds a robust pathway for accurately classifying all levels of diabetic retinopathy with improved generalization to unseen data and thus increased clinical reliability.

#### 2.3.4 Transformer encoder for global context

After extracting features from convolution for this purpose, the Transformer encoder layers were added to capture long-range dependency structures and permit global contextual information in retinal fundus images (33). Convolutional layers, as mentioned, are good at local texture extraction and relatively poor at relationship modeling over distances. This is where Transformers come in handy because they apply self-attention to the whole feature sequence, thereby making it possible for the network to learn local features and global ones as well. Given a reshaped feature map *F* ∈ ℝ^*N* × *C*^, where *N* = *H* × *W* is the flattened spatial dimension and *C* is the number of channels, the multi-head self-attention (MHSA) computes attention across all spatial locations. For each attention head (34), the query *Q*, key *K*, and value *V* matrices are obtained via learned linear projections:


$$\begin{array}{l} Q\;=\;F\;W_{Q},\;K\;=\;FW_{K},\;V\;=\;FW_{V} \end{array}$$ (13)

$$\begin{array}{l} Attention\left(Q,\;K,\;V\right)=\;softmax\left(\frac{QK^{T}}{\sqrt{d_{k}}}\right)V \end{array}$$ (14)

Where *d*_*k*_ is the dimension of the key vectors. The multi-head attention concatenates the outputs of all the heads and applies a final linear transformation, allowing the model to focus on information from different representation subspaces simultaneously. The operation of multi-head self-attention (MHSA) is followed by a feed-forward network (FFN) that is applied independently to each position and consists of two fully connected layers with a ReLU activation in between (35), and residual connections and layer normalization are employed after both the attention block and the FFN stabilize training and improve gradient flow:


$$\begin{array}{l} FFN\left(x\right)=\;W_{2}ReLU\left(W_{1}x\;+\;b_{1}\right)+\;b_{2} \end{array}$$ (15)

$$\begin{array}{l} x^{\prime }\;=\;LayerNorm\left(x\;+\;MHSA\left(x\right)\right)\;,\\ {\;x}^{\prime \prime }=\;LayerNorm\left(x^{\prime }+\;FFN\left(x^{\prime }\right)\right) \end{array}$$ (16)

By incorporating Transformer encoders (34), the model will learn the interaction between remote retinal regions, as in the spatial relation between the microaneurysms, hemorrhages, and exudates. The combination of the local convolutional features and global attention improves the detection of complex patterns across the retina by the network, which leads to better classification across all the severities of diabetic retinopathy. The Transformer encoder forms a hybrid CNN-Transformer architecture together with CBAM and DropBlock, which are complementary methods of fine-grained local feature extraction and broad global context understanding, reinforcing generalization and robustness on retinal datasets.

#### 2.3.5 Class balancing and weighted loss

The data for diabetic retinopathy can often be skewed, where the examples for some severity levels (e.g., Proliferative_DR or Mild) are way less than for the others (36). Training on these imbalanced data may result in skewed predictions toward the majority class. To rectify this, we computed the model loss such that a greater penalty was incurred for the wrong classification of the underrepresented classes to achieve a balanced learning for all severity levels. Given a dataset with *C* classes and *N* total training samples, let *n*_*c*_ denote the number of samples in class *c*, the class weight for class *c* is computed. The categorical cross-entropy loss during the training of a sample with a true label *y* ∈ {0, 1}^*C*^ and predicted probability ŷ ∈ {0, 1}^*C*^ is adjusted by the introduction of class weight.


$$\begin{array}{l} w_{c}=\frac{\;N}{\left(C\;*\;n_{c}\right)} \end{array}$$ (17)

$$\begin{array}{l} L=\;\overset{C}{\underset{c=1}{\sum }}w_{c}y_{c}\log\left(\;{\;ŷ}_{c}\right) \end{array}$$ (18)

The application of this weighting has the effect that the mistakes of the minority classes will have a greater impact on the total loss, thus making the network learn the common features of all classes (37). As a part of the strategy to deal with the class imbalance problem, in addition to the weighted loss, data augmentation was also used rather extensively. Among the augmentation methods were random rotations, brightness adjustments, zooming, and horizontal flips, which together resulted in creating more samples for the minority classes effectively. The combination of class-weighted loss and augmentation allowed the model to spread the learning evenly over the severity levels, hence making it possible for each class to have a similar representation during training. This method not only leads to a better classification accuracy but also makes the convergence process more stable and the generalization on previously unseen retinal images better.

#### 2.3.6 Grad-CAM for model interpretability

For the reason of clinical interpretability and visualization of retinal regions influencing model prediction, we incorporated Grad-CAM into our framework (38). Upon applying Grad-CAM to highlight the regions of the input image deemed most important for the predicted class, the clinicians may obtain insight into the decision-making process of the model. For the given input image *A*^*k*^∈ ℝ^*H*× *W*^ being the feature maps from the last convolutional layer *k*, where as *y*^*c*^ is the score for class c before softmax activation (39). The importance weight $\alpha_{k}^{c}$ for feature map *k* is computed by global average pooling of the gradients of *y*^*c*^ concerning *A*^*k*^. The Grad-CAM heatmap $L_{Grad-CAM}^{c}$ is thereby obtained with a weighted combination of feature maps followed by a ReLU activation:


$$\begin{array}{l} \alpha_{k}^{c}=\;\left(\frac{1}{\left(H\;\cdot W\right)}\right)\overset{H}{\underset{i=1}{\sum }}\overset{W}{\underset{j=1}{\sum }}\frac{\partial y^{c}}{\partial A_{ij}^{k}} \end{array}$$ (19)

$$\begin{array}{l} L_{Grad-CAM}^{c}=ReLU\;\left(\underset{k}{\sum }\alpha_{k}^{c}A^{K}\right) \end{array}$$ (20)

Features with a positive influence on class predictions are highlighted. The resultant heat map is then upscaled to the input image resolution and laid on the original image for interpretative purposes. Clinically relevant areas in microaneurysm, hemorrhages, and exudates are then visualized by the model through Grad-CAM as an explanation of its predictions, thereby supporting clinical validation and indicating possible failure cases where the network might be dependent on some irrelevant region (40). The application of Grad-CAM in conjunction with the CBAM and Transformer modules further enhances interpretability because the CBAMs supply attention maps, and the global context from Transformers reinforces the regions highlighted in the Grad-CAM visualizations.

### 2.4 Evaluation model

In the grading of diabetic retinopathy (DR), images generally fall into one of five severity categories: no DR, Mild DR, Moderate DR, Severe DR, and Proliferative DR. No DR here indicates that the retina appears normal and there are no pathological changes, whereas Mild DR indicates early-stage changes such as few microaneurysms or just minor hemorrhages. Moderate DR indicates more widespread pathological changes such as multiple hemorrhages or small exudates. Severe DR indicates considerable retinal damage with diffuse hemorrhages and, of course, vascular lesions; Proliferative DR describes the final stage, which is marked by abnormal neovascularization that can potentially lead to the loss of vision.

Evaluation metrics provide guarantees on the efficiency with which the model can perform on the test classes. Accuracy refers to the ratio of correctly classified images over the total number of images. Precision for a class tells us how many of the predicted cases for that class were correct. Sensitivity (Recall) measures the ability of the model to detect all true cases of a class, while specificity measures how well the model can identify that class from others. F1-score provides a trade-off between precision and sensitivity and is specifically important for the classes that are least common, like Proliferative DR.

For instance, a model may have a high positive predictive value but a low sensitivity for Proliferative DR, meaning that when it predicts this class, it is usually correct but will miss many true cases. Conversely, Mild DR is often more difficult to detect; hence, the importance of the F1-score to verify that mild cases are indeed detected, while minimizing false positives. The relationship between the metrics and each DR severity class is summarized in the Table 3 below.


$$\begin{array}{l} Accuracy=\frac{TP+TN}{TP+TN+FP+FN} \end{array}$$ (21)

$$\begin{array}{l} Precision=\frac{TP}{TP+FP} \end{array}$$ (22)

$$\begin{array}{l} Recall=\frac{TP}{TP+FN} \end{array}$$ (23)

$$\begin{array}{l} F_{1}-Score=2\frac{Precision\times Sensitivity}{Precision+Sensitivity} \end{array}$$ (24)

**Table 3:** Diabetic retinopathy levels and the importance of assessment measures.

**DR class**	**Description**	**Relevance of metrics**
No_DR	Normal retina	Accuracy and specificity show how well normal cases are correctly identified
Mild DR	Early, subtle changes	Precision, sensitivity, and F1-score evaluate detection of mild cases
Moderate DR	Intermediate severity	Metrics assess balance of correct detection vs. misclassification
Severe DR	Extensive retinal damage	Sensitivity and F1-score ensure severe cases are captured
Proliferative DR	Advanced stage with neovascularization	Precision and sensitivity indicate ability to detect high-risk cases

Finally, 95% confidence intervals for accuracy were estimated using the standard error of the proportion to quantify the statistical reliability of results:


$$\begin{array}{l} \;SE\;=\;sqrt\left(Accuracy\;*\;\frac{\left(1\;-\;Accuracy\right)}{N}\right),\\ CI_{95}\%\;=\;Accuracy\;\pm \;1.96\;\cdot SE \end{array}$$ (25)

### 2.5 Computing environment

All the trials were conducted with the help of Visual Studio Code (VS Code) on Windows 11 Pro. The system on which training and evaluation were performed had an Intel Core i7-12700K processor, 16GB RAM, and NVIDIA RTX4060ti GPU.

## 3 Experimental results

The proposed model was evaluated using fundus images from the APTOS 2019 and DDR datasets. This section provides a brief overview of the results obtained from the proposed CheXNet_CBAM model for classifying fundus images into five categories: no diabetic retinopathy (DR), mild DR, moderate DR, severe DR, and proliferative DR. Each training experiment included five different transfer learning models: CheXNet, DenseNet121, MobileNetV2, VGG19, and ResNet50.

The CheXNet_CBAM model was created based on DenseNet121 backbone architecture integrated with CBAM attention mechanism and Transformer Encoder layer for enhancing attention capability of the model toward critical features in medical images. To make it compatible with the pretrained models, images were resized to 224 × 224 pixels. The batch size was 16 and the epoch number was set to 50 for efficient learning from the data. An 80-10-10 split: for training, validation, and testing, was then performed to provide fair evaluation with ImageDataGenerator used for image rescaling (rescale 1/255) for training stability.

This is in comparison to some other deep learning models, for instance: MobileNetV2, VGG19, ResNet50, and standard DenseNet121, which were also trained based on the same core settings (image size, batch size, and epochs) with some techniques such as Dropout introduced to aid stability. However, CheXNet_CBAM incorporates the CBAM attention module, Transformer layer, and DropBlock2D to enhance feature focus while reducing overfitting. The means of the performance evaluation included test accuracy, confusion matrices, and 95% confidence intervals to conduct a reliability analysis of the results.

### 3.1 Comparing the performance of the proposed model, CheXNet_CBAM, with baseline models in diabetic retinopathy classification (APTOS 2019)

**Figure 5:**
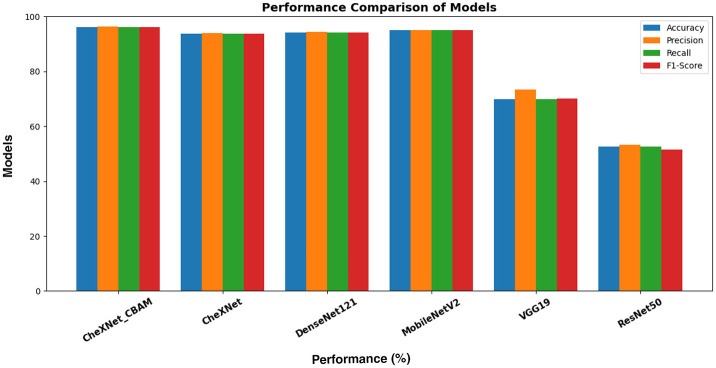
Comparison of performance metrics of different models on the APTOS 2019 dataset.

Table 4 presents the performance of the proposed CheXNet_CBAM model compared to various baseline models when applied to fundus images from the APTOS 2019 dataset for diabetic retinopathy (DR). The CheXNet_CBAM model achieved an accuracy of 96.12%, a precision of 96.30%, and an F1 score of 96.08%, outperforming the other models. The CheXNet model without CBAM achieved an accuracy of 93.80%, demonstrating the effectiveness of the proposed model and the idea of adding a CBAM layer to the CheXNet model, which significantly increased the performance of the CheXNet_CBAM model in diabetic retinopathy detection. Meanwhile, the MobileNetV2 model ranked second in diabetic retinopathy detection with an accuracy of 95.02% and an F1 score of 95.01%. The DenseNet121 model also achieved an accuracy of 94.24%. While VGG19 and ResNet50 performed the worst among the models in their ability to detect diabetic retinopathy, with VGG19 achieving an accuracy of 69.99%, ResNet50 ranked last with an accuracy of 52.71%. Figure 5 illustrates the model's effectiveness.

**Table 4:** Model's performance analysis using fundus images from the APTOS 2019 dataset.

**Model**	**Accuracy (%)**	**Precision (%)**	**Recall (%)**	**F1-Score (%)**
**CheXNet_CBAM**	96.12	96.30	96.12	96.08
**CheXNet**	93.80	93.96	93.79	93.75
**DenseNet121**	94.24	94.28	94.24	94.19
**MobileNetV2**	95.02	95.03	95.02	95.01
**VGG19**	69.99	73.37	69.96	70.00
**ResNet50**	52.71	53.17	52.70	51.53

Bold values highlight the best-performing model (CheXNet_CBAM), achieving 96.12% accuracy on APTOS.

**Figure 6:**
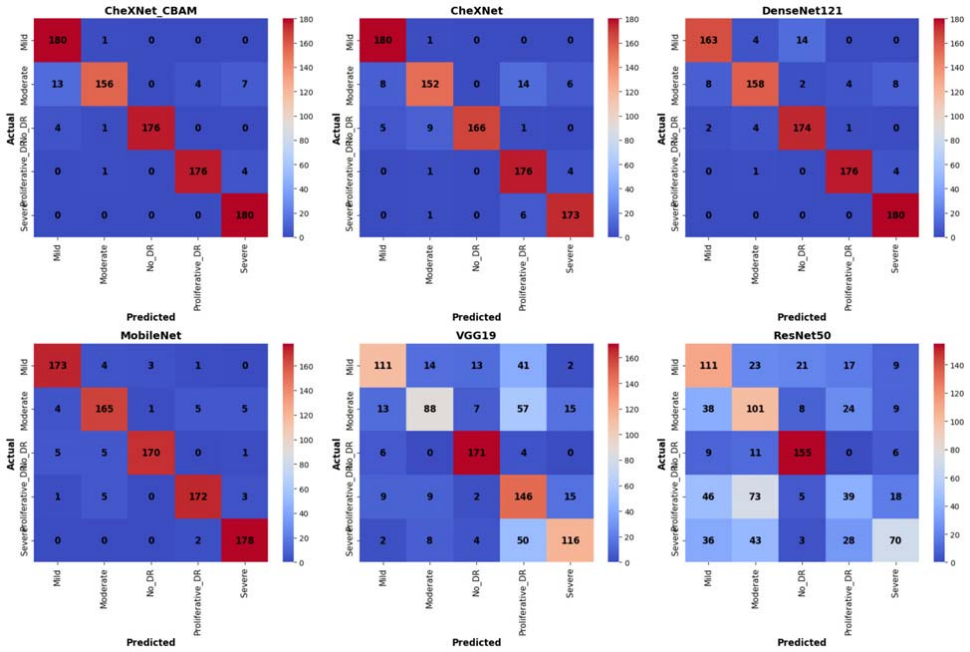
Confusion matrix of learning models using the APTOS 2019 dataset for diabetic retinopathy detection.

The confusion matrices in Figure 6 demonstrate the superior diagnostic accuracy of the proposed CheXNet_CBAM model compared to other models, achieving high diagnostic accuracy across all five disease categories (mild, moderate, non-diabetic retinopathy, proliferative, and severe). The model demonstrated exceptional ability to differentiate diabetic retinopathy cases, with significantly lower false positives and false negatives compared to CheXNet, DenseNet121, MobileNetV2, VGG19, and ResNet50. This significant performance improvement is attributable to the integration of the CBAM attention mechanism (convolutional block attention module) into the CheXNet architecture, which enables the model to more accurately focus on critical regions in retinal images containing signs of diabetic retinopathy.

The evaluation results of the proposed CheXNet_CBAM model by category, shown in Table 5, demonstrated exceptional performance in diagnosing all stages of diabetic retinopathy. It achieved a perfect precision of 100% in identifying cases (No_DR) with a high recall rate of 97.24%, demonstrating its excellent ability to avoid misdiagnosis of benign cases. The model also demonstrated excellent balance in diagnosing different disease stages, achieving high F1 rates ranging from 92.04% for moderate cases to 97.51% for proliferative cases. It also achieved outstanding performance in diagnosing severe cases with a perfect recall rate of 100% and a precision of 94.24%. These results confirm the model's high ability to accurately distinguish between different disease stages without missing any disease (especially severe and mild), while minimizing misdiagnosis rates.

**Table 5:** Performance evaluation of the CheXNet_CBAM model on the APTOS 2019 dataset, by category.

**Class**	**Precision (%)**	**Recall (%)**	**F1-Score (%)**
**Mild**	91.37	99.45	95.24
**Moderate**	98.11	86.67	92.04
**No_DR**	100.00	97.24	98.60
**Proliferate_DR**	97.78	97.24	97.51
**Severe**	94.24	100.00	97.04

Bold values indicate exceptional class-wise performance, including 100% precision for No_DR and 100% recall for Severe.

### 3.2 Comparing the performance of the proposed CheXNet_CBAM model with baseline models in detecting diabetic retinopathy using the DDR dataset

**Figure 7:**
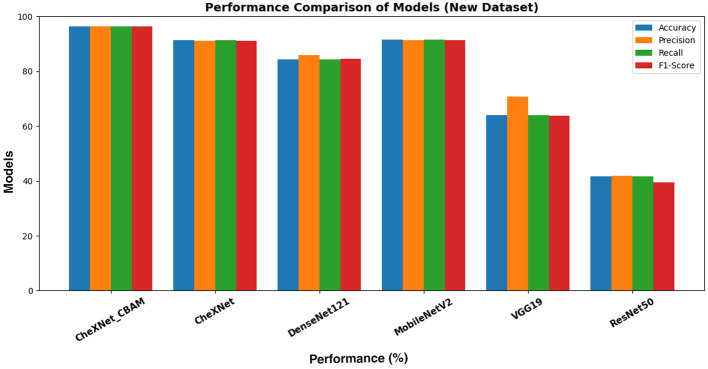
Comparison of performance metrics of different models on the DDR dataset.

Table 6 and Figure 7 provides a comprehensive overview of the performance of the proposed CheXNet_CBAM model compared to other baseline models when analyzing fundus images for diabetic retinopathy detection using the DDR dataset. The proposed model outperformed other models in detecting diabetic retinopathy with an accuracy rate of 96.33%, a precision rate of 96.29%, and an F1 score of 96.30%. The CheXNet model without CBAM achieved an accuracy of 91.35% and a precision of 91.18%. MobileNetV2 ranked second with an accuracy of 91.51% for detecting diabetic retinopathy. DenseNet121 and VGG19 achieved an accuracy of 84.42 and 64.06%, respectively. ResNet50 performed the worst among the models in detecting diabetic retinopathy with an accuracy of 41.72%.

**Table 6:** Model's performance analysis using fundus images from the DDR dataset.

**Model**	**Accuracy (%)**	**Precision (%)**	**Recall (%)**	**F1-Score (%)**
**CheXNet_CBAM**	96.33	96.29	96.33	96.30
**CheXNet**	91.35	91.18	91.35	91.10
**DenseNet121**	84.42	85.80	84.43	84.61
**MobileNetV2**	91.51	91.27	91.51	91.35
**VGG19**	64.06	70.85	64.05	63.74
**ResNet50**	41.72	41.89	41.71	39.46

Bold values highlight the best-performing model (CheXNet_CBAM), achieving 96.33% accuracy on DDR.

ResNet50 performance was low relatively with the APTOS-2019 and DDR datasets. Though the ResNet-based models have been consistently strong in diabetic retinopathy studies, their performance under hyperparameter tuning, training duration, and dataset-specific optimization is very dependent. For conducting our experiments, we had all baseline models under same conditions to maintain a fair and unbiased comparison. One reason for the poor performance of ResNet50 is its restricted use of features and lack of pronounced attention mechanisms, both of which are vital for picking up small and scattered retinal lesions. DenseNet-based architecture, on the contrary, even more so when enhanced with CBAM, permits a richer aggregation of multi-scale features and greater attention paid to the clinically significant areas. The factors combined, besides the proposed CheXNet_CBAM model's robustness and generalization capability across different fundus image datasets, have made it possible to further emphasize performance gap.

**Figure 8:**
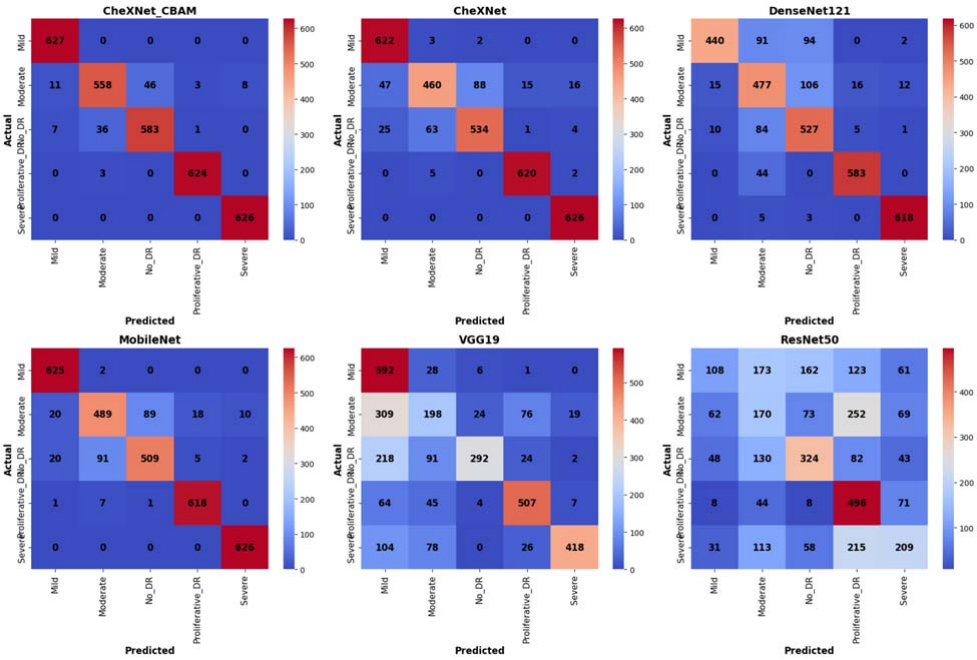
Confusion matrix of learning models using the DDR dataset for diabetic retinopathy detection.

Exceptional performance was achieved by the proposed CheXNet_CBAM model, which was presented in Figure 8 and unambiguously surpassed all other models in diagnosing diabetic retinopathy using the DDR dataset. The confusion matrix showed a perfect distribution of values for each class with the highest accuracy for the correct classification achieved. The main diagonal of the confusion matrix had the following values: 626 for severe cases, 634 for proliferative cases, 583 for non-proliferative cases, 558 for moderate cases, and 628 for mild cases. The CheXNet model made more misclassifications than the other models and those misclassifications were especially between adjacent classes. MobileNet and DenseNet121 had a relatively good performance, but their classifications overlapped to some extent. VGG19 and ResNet50, on the contrary, performed very poorly with misclassifications being widely spread across the classes. This clearly indicates the superior performance of the proposed CheXNet_CBAM model, which integrates the CBAM attention mechanism in overcoming high diagnostic accuracy and low error rates.

The CheXNet_CBAM model demonstrated exceptional ability to detect both mild and severe cases of diabetic retinopathy, achieving a perfect recall rate of 100% for both categories, as shown in Table 7. In severe cases requiring urgent medical intervention, it achieved an extremely high precision of 98.74%, detecting all cases, while in mild cases requiring early follow-up, its precision reached 97.21%. Severe proliferative cases were treated with exceptional precision, achieving an accuracy rate exceeding 99.36% across all metrics. Even in more complex cases, such as moderate and benign cases, the model maintained a strong performance exceeding 90%, confirming its ability to distinguish between all stages of the disease accurately. This balanced performance makes it a reliable medical tool that clinicians can rely on to make accurate and informed treatment decisions.

**Table 7:** Performance evaluation of the CheXNet_CBAM model on the DDR dataset, by category.

**Class**	**Precision (%)**	**Recall (%)**	**F1-Score (%)**
**Mild**	97.21	100.00	98.58
**Moderate**	93.47	89.14	91.25
**No_DR**	92.69	92.98	92.83
**Proliferate_DR**	99.36	99.52	99.44
**Severe**	98.74	100.00	99.37

Bold values indicate perfect recall rates (100%) for Mild and Severe classes, as well as exceptional performance for Proliferate_DR.

### 3.3 Statistical analysis

The statistical analysis in Table 8 reveals a clear performance hierarchy, with the CheXNet_CBAM model outperforming with an accuracy of 96.12%. CheXNet_CBAM shows a statistically significant improvement of 2.32 percentage points due to the integration of CBAM, compared to the CheXNet model with non-overlapping confidence intervals (95.23, 96.98) vs. (92.75, 94.82). The 1.10 percentage point difference represents a significant but smaller gap with the MobileNetV2 model, with slightly overlapping confidence intervals indicating moderate statistical significance. The huge performance gaps 26.13% vs. VGG19, 43.41% vs. ResNet50 show highly significant differences with non-overlapping confidence intervals.

**Table 8:** APTOS 2019 dataset—statistical performance analysis.

**Model**	**Accuracy (%)**	**Difference from the CheXNet_CBAM**	**95% confidence interval**
**CheXNet_CBAM**	96.12	0.0	(95.23, 96.98)
**MobileNetV2**	95.02	−1.10	(94.05, 95.97)
**DenseNet121**	94.24	−1.88	(93.21, 95.25)
**CheXNet**	93.80	−2.32	(92.75, 94.82)
**VGG19**	69.99	−26.13	(67.89, 72.07)
**ResNet50**	52.71	−43.41	(50.12, 55.28)

Bold values indicate the CheXNet_CBAM model as the reference baseline with the best performance on APTOS.

Improved Performance Gaps: the DDR dataset shows more pronounced performance differences, as shown in Table 9, with CheXNet_CBAM achieving 96.33% accuracy and larger gaps compared to the other models. The 4.98 percentage point improvement between CheXNet_CBAM and CheXNet is highly statistically significant with completely non-overlapping confidence intervals of (95.46, 97.18) vs. (90.18, 92.50). The 4.82 percentage difference between CheXNet_CBAM and MobileNetV2 demonstrates clear statistical significance. The 11.91 percentage point difference is statistically significant with non-overlapping confidence intervals. The 11.91 percentage point difference demonstrates statistical significance with non-overlapping confidence intervals between CheXNet_CBAM and DenseNet121.

**Table 9:** DDR dataset—statistical performance analysis.

**Model**	**Accuracy (%)**	**Difference from the CheXNet_CBAM**	**95% confidence interval**
**CheXNet_CBAM**	96.33	0.0	(95.46, 97.18)
**MobileNetV2**	91.51	−4.82	(90.35, 92.65)
**CheXNet**	91.35	−4.98	(90.18, 92.50)
**DenseNet121**	84.42	−11.91	(82.95, 85.87)
**VGG19**	64.06	−32.27	(61.78, 66.32)
**ResNet50**	41.72	−54.61	(38.65, 44.77)

Bold values indicate the CheXNet_CBAM model as the reference baseline with the best performance on DDR.

The statistical analysis results provide strong evidence of the superiority of the proposed CheXNet_CBAM model, achieving high performance on both datasets and demonstrating significant differences from all competing models. Clinical reliability, thanks to precise confidence intervals, ensures predictable performance, with the model's generalizability. Overall, the inclusion of confidence intervals provides clinically meaningful effect size interpretation and supports the reliability and generalizability of the proposed CheXNet_CBAM framework.

## 4 Discussion

### 4.1 Comparison with other studies

The meaning of the trend is the development of the classification of diabetic retinopathy that is prevalent in its various features, from methods, from feature fusion to attention-based techniques. For instance, RT2Net integrated fundus and vascular branch networks (41) and achieved 88.2% on EyePACS and 85.4% on APTOS, while few-shot learning with Siamese neural networks and pre-trained models such as VGG16 and ResNet50 (42) reported more modest results (80%−81%).

Such results were shown by EfficientNetB3-enhanced models with SE processing blocks (43), which achieved an 88.44% accuracy rate. Attention-driven methods, such as MSCAS-Net (44), have also greatly raised this performance level to an astounding 93.8% on the APTOS database, surely proving how important multi-scale and attention fusion are in performance improvement. Therefore, it is evident that the introduction of attention mechanisms and feature-level fusion is more advantageous when compared with standard transfer learning techniques. Other studies have concentrated on interpretability and hybrid learning for the sake of accuracy vs. clinical applicability. With an amazing 94.64% on APTOS, the explanation AI is from ResNet-50 with SHAP (45) compared with traditional CNN attention frameworks such as MSRAB + CrAB (46), which achieved 88.31% on APTOS. Bayesian deep learning approaches (47) achieved the highest performance, with 94.23% accuracy using MC Dropout, emphasizing their strength in uncertainty-aware classification. Hybrid CNN–ViT models with interpretability tools such as LIME and Grad-CAM (48) also demonstrated high performance (93.01%), showing that combining convolutional and transformer-based features yields both strong results and interpretability.

In the meantime, hybrid strategies that fuse deep features with classical machine learning did not do that well, compared to the few, but for example, an SVM classifier combined with EfficientNetV2-S (49) worked at 91%, while the lightweight two-stage models (50) had an accuracy of 90.75%-which is just a marginal improvement. Swin-TransformerV2 with hybrid attention (51) showed performance with average accuracy (85.5%−87.9%), not so bad compared to CNN-driven models. Nonetheless, our proposed model, CheXNet_CBAM, which incorporates methods like DenseNet121 with CBAM, DropBlock2D, and a Transformer Encoder, attained 96.12% accuracy on APTOS and 96.33% accuracy on DDR-with the rest of the models competing with the Bayesian best ones.

The most salient points of improvement to CheXNet include the focus on clinically relevant retinal regions that was made possible with the incorporation of the convolutional block attention module (CBAM) within the model, DropBlock2D for greater regularization control and preventing any tendencies to overfit, and the use of a Transformer Encoder to capture contextual and long-range spatial dependencies in retinal images. Such a hybrid model stands to combine the strengths of contextualization with CNN-based feature extraction via attention mechanisms and transformers. Therefore, CheXNet_CBAM is not only better in terms of accuracy than most state-of-the-art methods, but also provides a sound and generalizable framework, making it a prime candidate for real clinical deployment. Table 10 shows a comparison of previous studies on the diagnosis of diabetic retinopathy.

**Table 10:** Comparison of the results of the proposed study with previous studies for the classification of diabetic retinopathy.

**Year**	**Reference**	**Dataset**	**Approach**	**Model used**	**Accuracy**
2024	(41)	EyePACS, APTOS-2019	Multi-view Joint Learning + Feature Fusion	RT2Net (Fundus + Vascular Branch Networks)	88.2% (EyePACS), 85.4% (APTOS-2019), AUC: 0.98 (EyePACS), 0.96 (APTOS)
2024	(42)	FGADR, APTOS-2019	Few-shot learning using similarity-based classification	Siamese Neural Network + Pre-trained models (VGG16, ResNet50, DenseNet121)	FGADR: 80% APTOS-2019: 81%
2025	(43)	APTOS-2019, IDRiD, Messidor-2	Transfer Learning + SE Block	EfficientNetB3 + SE	88.44%
2025	(44)	APTOS, DDR, IDRiD	Fine-grained + Attention Fusion	MSCAS-Net (Swin Transformer + Multi-Scale Attention)	93.8% (APTOS), 89.8% (DDR), 86.7% (IDRiD)
2025	(45)	APTOS-2019, EyePACS, DDR, IDRiD, SUSTech-SYSU	Transfer Learning + Explainable AI	ResNet-50 + SHAP	94.64% (APTOS), 86.36% (EyePACS), 84.23% (DDR), 82.79% (IDRiD), 85.65% (SUSTech-SYSU)
2025	(46)	APTOS-2019, DDR	Multi-scale Residual + Cross-Attention	CNN + MSRAB + CrAB	APTOS-2019 :88.31% DDR : 84.15%
2025	(47)	APTOS-2019,	Bayesian Deep Learning	DenseNet-121 + Bayesian (MC Dropout, MFVI, Det.)	94.23%
2025	(52)	DDR, IDRiD	Dual-stage grading + Feature Collaboration	XE-Net + MFC-Net (lesion + vascular)	IDRiD:91.26 DDR: 89.24
2025	(53)	APTOS-2019, MosMedData CT	Privacy-Preserving Federated Learning + Homomorphic Encryption	EfficientNet-B0 + PPFLHE Framework	83.19% (APTOS), 81.27% (MosMedData)
2025	(48)	APTOS-2019	Hybrid CNN + ViT + Explainable AI	ResViT FusionNet (ResNet50 + ViT + LIME + Grad-CAM)	93.01%
2025	(51)	DDR, APTOS-2019, Clinical dataset	Multi-branch Fine-Grained Classification + Hybrid Attention	Swin-TransformerV2 + Multi-Branch + Category Attention	DDR: 87.9% APTOS-2019: 85.5% Clinical data: 77%
2025	(49)	APTOS 2019	Transfer learning with deep learning for multi-stage DR classification	ResNet_101, DenseNet_201, EfficientNet_b0 (best: EfficientNet_b0)	91%
2025	(50)	APTOS 2019	Two-stage deep learning: Stage 1 – DR detection (healthy vs diseased), Stage 2 – DR severity classification using transfer learning	Lightweight multi-deep learning framework (custom two-stage model)	90.75%
**2025**	**Proposed model**	**APTOS 2019, DDR**	**DenseNet121 for integrated with CBAM Block, DropBlock2D Layer, Transformer Encoder**	**CheXNet_CBAM**	**APTOS: 96.12%, DDR: 96.33%**

Bold values highlight the proposed model results (96.12% on APTOS and 96.33% on DDR), representing the novel contributions of this study.

Recent multicenter studies have demonstrated the effectiveness of deep convolutional and attention-based models for diabetic retinopathy screening across heterogeneous populations and imaging devices. While these works primarily focus on large-scale performance benchmarking, limited attention has been given to integrating lightweight attention mechanisms with dense feature reuse and explicit visual interpretability. In contrast, the proposed CheXNet_CBAM framework emphasizes both classification performance and clinical transparency through Grad-CAM visualization, while maintaining robustness across multiple public datasets. This complementary focus distinguishes the present study and contributes incremental value to existing multicenter research.

### 4.2 Grad-CAM visualization of CheXNet_CBAM for diabetic retinopathy detection

**Figure 9:**
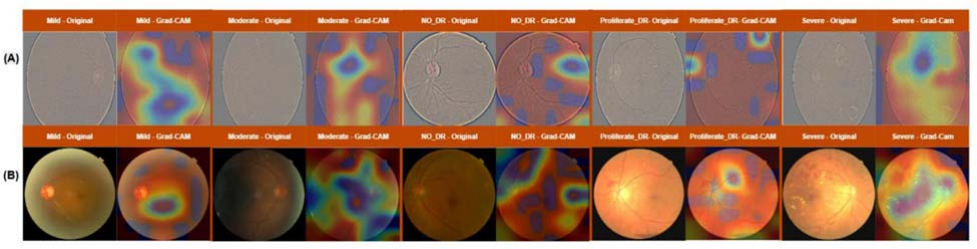
Grad-CAM Interpretive Maps of Diabetic Retinopathy Scores on **(A)** APTOS-2019 and **(B)** DDR Datasets.

Figure 9 presents Grad-CAM visualizations of the proposed CheXNet_CBAM model when applied to fundus images from the DDR and APTOS 2019 datasets, respectively. These visualizations provide insights into the regions of interest emphasized by the model during diabetic retinopathy (DR) detection, highlighting how the network interprets subtle to severe pathological changes.

In Figure 9A (APTOS 2019 dataset), the Grad-CAM output is compared to DDR and displays more pronounced and localized activations, which is probably due to the better image quality of the dataset. The network highlights the small changes around the macula in case of mild DR, but in the case of moderate DR, it reveals more accurately the presence of small hemorrhages and vascular malformations. In No_DR, the distributions of activations stay at the same low levels and are dispersed, which is an indication of a correct diagnosis. In proliferative DR and severe DR, the visualizations highlight pathological areas, including abnormal vascular growth and widespread hemorrhages, confirming the model's ability to identify important clinical features in advanced cases.

In the DDR dataset shown in Figure 9B, the Grad-CAM maps indicate that in case of Mild DR the model's attention is mostly given to the macular area where early retinal changes are taking place. During Moderate DR, the focus is progressively broader and includes microaneurysms and hemorrhages which are in places across retina. No_DR condition is characterized by faint and weak activations, in line with the lack of pathological conditions. On the contrary, the network highlights the areas of neovascularization very well for Proliferative DR, while for Severe DR it points to the areas of extensive bleeding and exudates, giving out intense activations all over the infected zones. Generally, these Grad-CAM results indicate that the suggested CheXNet_CBAM model not only provides better accuracy in classification but also gives clinically significant interpretation. The differences between DDR and APTOS 2019 datasets reveal the impact of dataset quality on the not so clear model visualization for DDR and the very clear attention maps for APTOS. The interpretability that comes with this model increase its acceptance in the field of eye care since it allows ophthalmologists to follow and even trust the network's decision-making process.

The Grad-CAM visualizations consistently highlight anatomically and clinically meaningful regions, including areas around the macula, optic disc, and vascular structures where diabetic retinopathy lesions are known to occur. These activation patterns align with established ophthalmic diagnostic criteria reported in the literature, supporting the clinical plausibility of the learned representations. Although quantitative expert annotation-based validation was not available for the utilized public datasets, the observed consistency across datasets and severity levels provides indirect evidence of interpretability reliability.

### 4.3 Integration of the proposed method into the hospital

The proposed CheXNet_CBAM framework is intended as a clinical decision-support system (CDSS) for diabetic retinopathy (DR) screening/triage, rather than as an autonomous diagnostic tool. In a hospital setting, the workflow would be integrated into routine ophthalmic imaging processes. Retinal fundus images would be acquired using non-mydriatic or mydriatic fundus cameras according to local clinical protocols and transferred to an on-premises, access-controlled inference server over the hospital intranet in standard formats (e.g., JPEG or PNG). Prior to inference, images would undergo automated preprocessing consistent with the training pipeline, including resizing to the model input resolution, applying the predefined normalization procedure, and applying an image-quality control step to identify ungradable or non-conforming images (with explicit criteria to be reported). Inference would be executed within a version-controlled software environment (e.g., Python with TensorFlow or PyTorch) using pinned library versions to improve reproducibility across deployments. The system would output both the predicted DR grade and Grad-CAM heatmaps highlighting image regions that most strongly influence the model output, and these outputs would be presented to clinicians via a graphical user interface to support review and documentation. The predictions and justification would be reviewed by the clinicians and they would be able to confirm, override or ask for an image to be re-taken depending on the local practice; the clinical responsibility stays with the physician who finally decides about the patient's management. A structured onboarding would be provided to the users covering the intended use, the interpretation of outputs, and the limitations of AI-assisted screening. Quality assurance would include performance monitoring at periodic intervals using predefined validation cases and acceptance criteria, logging of predictions and model versions for auditability, and targeted review of attribution maps to flag implausible patterns while noting that saliency methods do not establish causal relevance. The appropriate response to suspected performance degradation (e.g., due to acquisition changes) would include manual review and, if justified, a governed model update process with re-validation prior to redeployment. Although evaluation through public datasets can demonstrate generalization in controlled conditions, device- and site-specific external validation is still required to ensure safe integration into heterogeneous real-world screening workflows.

### 4.4 Limitations of this study

Regardless of the stated effectiveness, the CheXNet_CBAM model that has been suggested has limitations that the researchers consider very important. To begin with, the model was trained and tested on datasets that are publicly available which could be a reason for not reflecting completely the heterogeneity and case-mix of real-world patient populations met in the clinics. The differences in age distribution, disease prevalence in different areas, and other characteristics of the population may lead to the model being less accurate and less reliable when used in hospital settings different from those of the assessed datasets. Second, the assessment was retrospective and based on pre-existing datasets; no prospective workflow integration or evaluation of decision impact was performed. Consequently, downstream clinical outcomes—such as the appropriateness and timeliness of referral pathways—could not be assessed. Prospective, site-specific external validation in real clinical environments is therefore necessary to characterize clinical utility and safety. Third, as with AI-assisted screening systems generally, potential safety risks remain when translating the approach into practice, including misclassification of disease severity and inappropriate triage of higher-risk cases. The proposed method is intended to function as a decision-support system rather than an autonomous diagnostic tool, and clinical responsibility remains with qualified clinicians who make final management decisions. Although Grad-CAM heatmaps can provide qualitative attribution cues that may support clinical review, saliency-based explanations do not guarantee faithfulness and should not be treated as a standalone indicator of prediction reliability. Finally, deployment depends on operational infrastructure and governance, including secure integration into clinical workflows, technical support, fallback procedures for manual screening, and ongoing user training. Because data-driven models may also reflect dataset and labeling biases, ongoing monitoring, periodic validation, and recalibration as needed are required to maintain performance across evolving imaging devices, acquisition protocols, and clinical practices. Collectively, these considerations may hinder translation unless addressed through rigorous external validation and deployment governance.

## 5 Conclusion and further work

This study presents CheXNet_CBAM, a model for classifying retinal fundus images by diabetic retinopathy (DR) severity, including a No DR class. The model was trained and evaluated on the APTOS 2019 and DDR datasets. The performance was evaluated as per the reported experimental protocol against CheXNet/DenseNet121 and four more deep learning baselines. CheXNet_CBAM, with a test accuracy of 96.12% on APTOS 2019 and 96.33% on DDR, surpassed the accuracy of all evaluated baselines on the same splits. In the future, the use of other attention features and image processing techniques to enhance the system's resistance to low-grade or ungradable fundus images may be studied. Moreover, in the case of data availability and proper validation, a multimodal extension of CheXNet_CBAM might be investigated to add more clinical inputs for DR severity grading.

Despite these encouraging results, the findings should be interpreted within the context of dataset-centered evaluation. The model was trained and tested on publicly available datasets, which may not fully capture the heterogeneity of real-world clinical environments, including variations in patient demographics, imaging devices, and acquisition protocols. Therefore, while the results indicate strong potential, further validation is required before clinical deployment.

Future work will focus on several concrete and actionable directions. First, multicenter and multi-device validation studies will be conducted to assess generalizability across diverse populations and imaging conditions. Second, prospective clinical studies will be designed to evaluate real-world performance, safety, and workflow integration under physician supervision. Third, improvements to preprocessing and attention mechanisms will be explored to enhance robustness against low-quality fundus images, while minimizing the exclusion of samples. Finally, multimodal extensions incorporating complementary clinical information, such as patient demographics or optical coherence tomography data, will be investigated further to improve diabetic retinopathy severity grading and clinical decision support.

## Data Availability

The original contributions presented in the study are included in the article/supplementary material, further inquiries can be directed to the corresponding author.
